# Association between Irrigation Fluids, Washout Volumes and Risk of Local Recurrence of Anterior Resection for Rectal Cancer: A Meta-Analysis of 427 Cases and 492 Controls

**DOI:** 10.1371/journal.pone.0095699

**Published:** 2014-05-13

**Authors:** Can Zhou, Yu Ren, Juan Li, Ke Wang, Jianjun He, Wuke Chen, Peijun Liu

**Affiliations:** 1 Department of Surgical Oncology, the First Affiliated Hospital, School of Medicine of Xi’an Jiaotong University, Xi’an, Shaanxi Province, China; 2 Department of Translational Medicine Center, the First Affiliated Hospital, Xi’an Jiaotong University, Xi’an, Shaanxi Province, China; Centre for Inflammation Research, United Kingdom

## Abstract

**Background:**

Rectal washout can prevent local recurrence after anterior resection of rectal cancer. Few studies have focused particularly on the association between irrigation fluids volume or agents and the risk of local recurrence after anterior resection of rectal cancer.

**Objective:**

To estimate the association between irrigation fluids types, volumes of rectal washout and risk of local recurrence after anterior resection for cancer.

**Data Sources:**

Relevant studies were identified by a search of *Medline, Embase, Wiley Online Library, China National Knowledge Infrastructure, Cochrane Oral Health Group Specialized Register, Wanfang databases and Google Website* from their inception until *October 18,2013*.

**Study Selection:**

Studies reporting the association between rectal washout types and volumes and risk of local recurrence after anterior resection for cancer were included.

**Interventions:**

Eligible studies used rectal washout. Control groups were defined as no washout.

**Study Appraisal and Synthesis Methods:**

Random-effects model were used to obtain summary estimates of RR and 95% CI, with Stata version *11* and RevMan *5.2.5* softwares used. The quality of report was appraised in reference to the MINORS item.

**Results:**

Of the *919* rectal cancer patients in 8 included studies, a total of 61(6.64%) cases of local recurrence were reported, with a pooled RR *0.51 (95%CI = 0.28–0.92, P = 0.03).* The *RRs 0.37* and *0.39* in normal saline and washout volume (*≥1500 ml* normal saline) subgroup, respectively, indicated that rectal washout with normal saline, or *≥1500 ml* in volume could significantly reduce local recurrence (LR) rate *(95% CI = 0.17–0.79, P = 0.01; 95% CI = 0.18–0.87, P = 0.02)* after anterior resection for cancer.

**Limitation:**

The included studies were non-randomized observational studies, with diversity of study designs.

**Conclusion:**

Rectal washout with normal saline alone can reduce the risk of local recurrence in patients with resectable rectal cancer, and 1.5 liters rectal washout in volume is recommended.

## Introduction

Post-operative local recurrence (LR) of rectal cancer may yield severe outcomes that are associated with severely disabling symptoms and difficult to treat [1]. The rate of LR is the highest in the first two years after anterior resection of rectal cancer [2]–[4], ranging from 3% to 50% [1], [5]–[7].

It was hypothesized one hundred years ago that “liberated cancer cells” may cause recurrence after surgery for rectal cancer [8], [9], and most surgeons today continue to avoid touching or manipulating a tumor excessively, so as not to spread malignant cells inside or outside the bowel. An accumulating number of studies have confirmed that free malignant cells are shed into the lumen of rectum [10]–[14], and that mechanical lavage or the tumoricidal agents contained in rectal washout has the potential to eradicate free malignant cells shed into the rectal stump[5], [14]–[16]. It also found that patients with rectal washout have a more favourable outcome than those without washout[3], [17]–[20]. Clinical evidence has also demonstrated that rectal washout is associated with reduced post-operative local recurrence [5], [21]–[27] and that the completeness of cleansing with irrigation fluid is volume-related [14], [28]. Nevertheless, few studies have focused particularly on the effects of the volume or agents contained in the employed irrigation liquid. These agents have inherent biological characteristics of tumoricidal efficacy, such as cetrimide, povidone-iodine, sodium hypochlorite and formalin[5], [16], [18], [29]–[33], and non-antineoplastic efficacy, such as normal saline [34].

Thus, at least two critical questions remain unanswered: i) whether association exists between irrigation fluid type and risk of local recurrence after anterior resection for cancer; ii) and whether washout volume influence the risk of local recurrence after anterior resection for cancer. These questions are important for both future research and current clinical practice.

This meta-analysis is conducted to comprehensively assess the overall evidence regarding local recurrence following rectal washout with different irrigation fluid types and washout volumes, by scrutinizing pertinent original research articles and analyzing the pooled data, with the aim of to provide meaningful clues for prevention of local recurrence after anterior resection in patients with rectal cancer.

## Methods

The a priori review protocol was registered and published in the International Prospective Register of Systematic Reviews (PROSPERO), with the registration number # CRD42013006467. This report complies with the preferred reporting items of PRISMA for systematic reviews and meta-analyses [35].

### Criteria for Considering Studies for this Review

We included case controlled studies that enrolled adult patients with available rectal washout data. There was no minimum trial duration. Eligible studies were defined as studies used any type of rectal washout (i.e. normal saline, cetrimide, povidone-iodine or formalin) after anterior resection for cancer, with the following criteria to meet: i) comparing rectal washout (WO) with no washout (NWO); ii) characterizing the surgery as anterior resection or sphincter-sparing surgery, or as laparoscopic or hand-assisted resection; iii) in which the outcome was a local or anastomotic recurrence of rectal cancer and LR was diagnosed by palpation, imaging, endoscopy, cytopathology or histopathology. Control groups were defined as there was no any type of washout during the same study period as the experimental group.

The primary outcome was the associations between irrigation fluids type and risk of local recurrence for rectal cancer after anterior resection because the choice of fluids type is increasingly seen as important by medical workers, especially surgeons. The primary outcome was based on palpation, imaging, endoscopy, cytopathology or histopathology. Secondary outcome was the association s between washout volumes and risk of local recurrence after anterior resection for rectal cancer, as there has no conclusion that how much rectal washout is appropriate reducing the risk of local recurrence after anterior resection for cancer.

### Search Methods for Identification of Studies

The meta-analysis was conducted according to the checklist of the Meta-Analysis of Observational Studies in Epidemiology groups[36]–[37]. *Medline, Embase, Wiley Online Library, Cochrane Oral Health Group Specialized Register and* other sources such as *Google Website* were searched from their inception until *October 18, 2013,* using the terms “rectal washout”, “rectal irrigation”, “rectal neoplasms”, “rectal surgery”, “rectal stump”, “anterior resection”, “local recurrence”, and “local failure”. Relevant articles, which evaluate the risk of local recurrence or anastomotic recurrence by comparing patients with and without rectal washout, were identified. Both free text search and MeSH search were employed. The reference lists of the pertinent articles were also considered.

### Selection of Studies

Two independent reviewers (Can Zhou and Yu Ren) blinded to the results of the other reviewer first screened all records at the title level. To enhance sensitivity, records were only removed if both reviewers excluded at the title level. The second level of review was at the abstract level followed by another round of review at the full-text level. All eligible studies were assessed a second time for relevance to ensure the objectivity of the review.

### Data Extraction and Quality Assessment

The information extracted from each publication, in the form of a table, included the following: name of the lead investigator, year of publication, primary end points, follow-up time, methods for assessment of end points, proportions of men and women, total number of subjects, person-years of follow-up, number of events, and RRs or hazard ratios with 95% CIs. To ascertain the validity of the eligible studies, the quality of each report was appraised in reference to the 12 item described in methodological index for non-randomized studies (MINORS) a quality assessment tool specifically designed to assess the methodological quality of non-randomized surgical studies [38]. Two reviewers (Can Zhou and Juan Li)independently scored all of these criteria on a scale ranging from 0 to 2, depending on whether the criterion was not reported (0), reported but inadequate (1), or reported and adequate (2). We added the criterion ‘randomization’ as both randomized and nonrandomized controlled studies were included, and scored 0 for nonrandomized studies and 2 for randomized studies. Scoring differences were discussed until consensus was reached. The total quality score ranges from 0 (low quality) to 26 (high quality). When there was disagreement it was resolved by discussion with corresponding author, via e-mail or personal interview.

### Assessment of Risk of Bias in Individual Studies

To assess heterogeneity[39]–[40], we used Begger’ Funnel Plot, Egger’s regression test, as well as Cochran’s heterogeneity statistics and Higgins I^2^ coefficient, a value that describes the percentage of variation across studies that are due to heterogeneity rather than chance, where I^2^ = 0% indicates no observed heterogeneity, with 25% regarded as low, 50% as moderate, and 75% as high. If notable heterogeneity was detected, a sensitivity analysis was performed for all studies to further investigate the study heterogeneity. Statistical significance for the interpretation of the Egger’s test was defined as *P<0.10*.

### Data Synthesis

Random-effects model was used to obtain summary estimates of RR and 95% CI. We performed the analysis using *Stata version 11* (StataCorp LP, College Station, Texas) and *RevMan 5.2.5* (Cochrane Collaboration, Oxford, United Kingdom) softwares. The results from the random-effects model were reported since there was some difference in heterogeneity across trials. RRs were used to measure the treatment effect as final values between the rectal washout (WO) and no rectal washout (NWO) groups. The value *RR<1* indicated a low risk of local recurrence and would be considered to be statistically significant if the 95% CI did not overlap 1. For all tests, a two-sided *p-value* below 0.05 was considered significant.

To validate the credibility of outcomes in this meta-analysis, sensitivity analysis was performed by sequential omission of each individual study using the *“metaninf” Stata* command.

## Results

### Results of the Search

**Figure 1:**
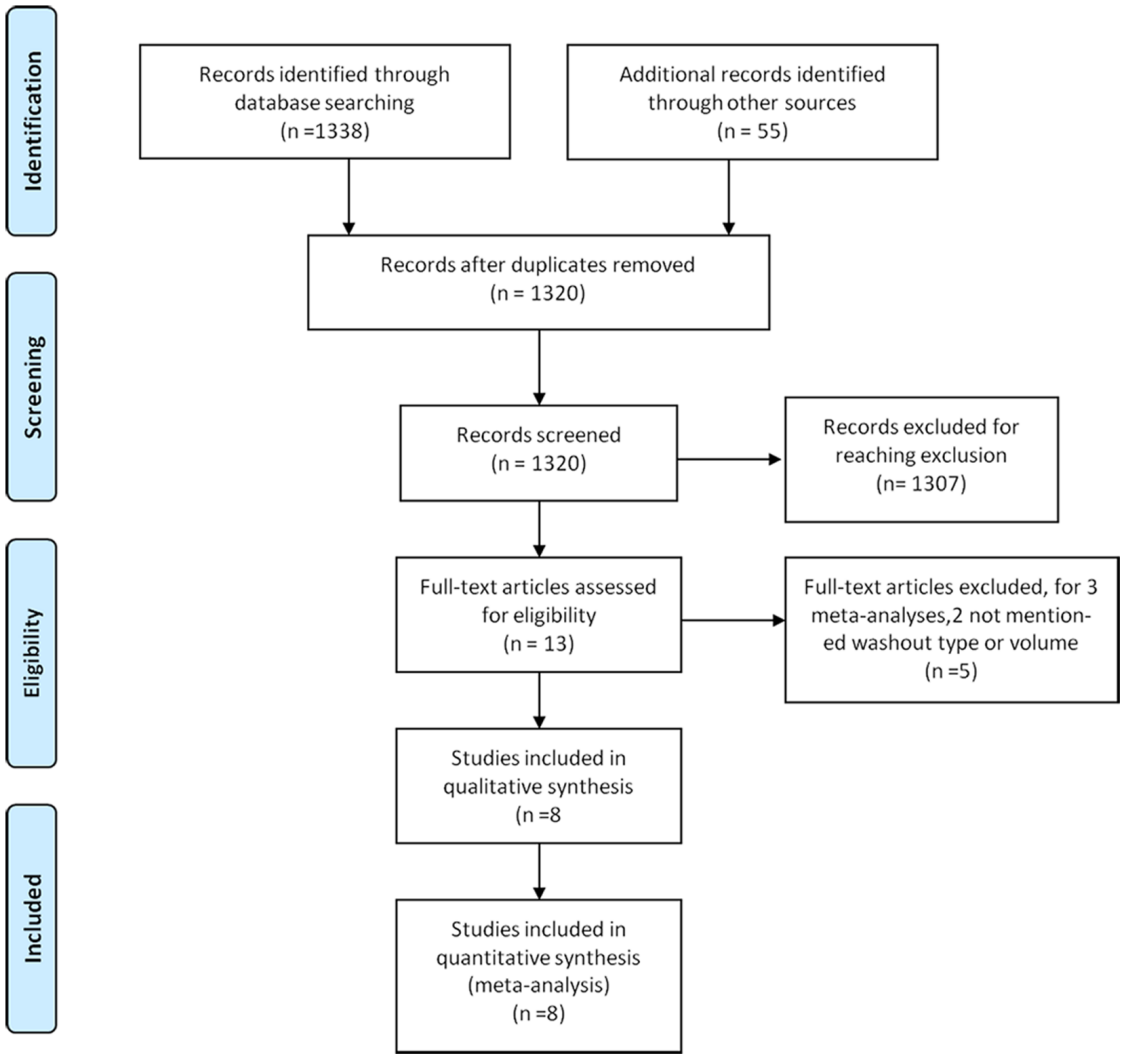
Flow diagram of study selection.

The steps of our literature search are shown in *figure 1*. We retrieved 1,393 reports (of which *1,338* through database searching, *55* through other sources) in our preliminary search. Of these, 7*3* were excluded for duplicated, *1,303* were excluded for meeting the exclusion criteria. Of the remaining *13* studies[5], [16]–[18], [20]–[24], [26], [34], [41], [42],*3* were excluded for meta-analyses [5], [26], [34], and 2 excluded for not mentioning irrigation types or washout volumes [41], [42]. Thus, 8 studies [17]–[19], [21]–[25] were included in this meta-analysis. The MINORS scores range from 16 to 20 points (*Table 1*). According to the quality criteria, all studies were moderate to high quality.

**Table 1:** Methodological quality assessment of the included studies.

Item#	First author							
	Fukuda [18]	Kawahara [22]	Nakano [23]	Shinto [24]	Xingmao [25]	Agaba [17]	Terzi [21]	Long [19]
Clearly stated aim	2	2	2	2	2	2	2	2
Inclusion of consecutive patients	2	2	2	2	2	2	2	2
Prospective collection of data	2	2	2	0	2	0	2	2
Endpoints appropriate to aim of study+ITT	2	1	2	2	2	2	2	2
Unbiased assessment of study endpoint(s)	2	2	2	2	2	2	2	2
Follow-up period appropriate to aim of study	2	2	0	2	2	2	1	2
Loss to followup<5%	2	2	0	2	2	2	2	2
Prospective calculation of study size	0	0	0	0	0	0	0	0
Adequate control group	2	2	2	2	2	2	2	1
Contemporary groups	2	2	2	2	2	2	2	2
Baseline equivalence of groups	2	2	2	2	2	2	2	2
Adequate statistical analyses	2	2	2	2	2	2	2	2
Randomization*	0	0	0	0	0	0	0	0
Total score	20	19	16	18	20	18	19	19

# 0 = not reported; 1 = reported but inadequate; 2 = reported and adequate;

*0 for nonrandomized studies and 2 for randomized studies; ITT: intention-to-treat.

### Study Characteristics

The characteristics of the eight included studies, which were performed in separate research centers and non-randomized controlled case-control ones, are shown in *Table 2*. A total of *919* patients were included in the meta-analysis, of whom *427 (46.5%)* underwent rectal washout and 492 (53.5%) did not have rectal washout during anterior resection for rectal cancer, with an overall LR rate of *6.64% (61/919)*. Of the eight studies, six were prospective studies, two were retrospective ones. Four studies published in English, were conducted in Turkey [21], United Kingdom [17], China [25] and the United States [19], respectively. The remaining four studies[18], [22]–[24] published in Japanese were conducted in Japan. The Japanese papers were analyzed with the help of a Japanese translator. Local recurrence was reported in all the eight studies and anastomotic recurrence was reported in six studies. Five studies used normal saline[18], [22]–[25] as the washout solution but with different washout volumes, ranging from 600 ml to 2000 ml, and the remaining three studies used 1% cetrimide [17] (500 ml), 5% povidone-iodine [21] (500 ml) and 1% formaline [19] (10∼20 ml), respectively.

**Table 2:** Characteristics and Demographics of Included Studies.

First author	Year	Type	Group	N	n	Mean Age	Women	Washout		Endpoints	DFU
							(%)	Type	Volume(ml)		(months)
Fukuda et al [^17^]	1991	P	*WO*	26	0	NS	42.3	Saline	600	LR, AR	48
			*NOW*	109	12	NS	40.4				48
Kawahara et al [^21^]	1998	P	*WO*	48	0	60.3	25	Saline	2000	AR	48
			*NOW*	52	3	60.2	40.4				48
Nakano et al [^22^]	2004	P	*WO*	34	1	63.3	32.4	Saline	2000	AR	NS
			*NOW*	35	3	64.2	28.6				NS
Shinto et al [^23^]	1996	R	*WO*	114	4	59.6	46.5	Saline	2000	AR	48
			*NOW*	80	9	61.8	52.5				48
Xingmao Z [^24^]	2013	P	*WO*	69	3	56.3	58	Saline	1500	LR, AR	48
			*NOW*	75	5	59	56				48
Agaba et al [^16^]	2004	R	*WO*	90	4	63	37.8	Cetrimide	500	LR	63
			*NOW*	51	3	61	40.4				63
Terzi C [^20^]	2006	P	*WO*	38	3	59.6	31.6	Povidone-iodine	500	LR, AR	36
			*NOW*	58	2	61.8	51.7				33
Long et al [^18^]	1989	P	*WO*	12	1	NS	NS	Formaline	10–20	LR	60
			*NOW*	28	8	NS	NS				60

R = retrospective; P = prospective nonrandomized; WO = washout group; NWO = no washout group; N: number of patients; n : number of events; NS = not stated; LR = local recurrence; AR = anastomotic recurrence; DFU = duration of follow-up.

### Association of Rectal Washout Solutions with Risk of Local Recurrence

**Figure 2:**
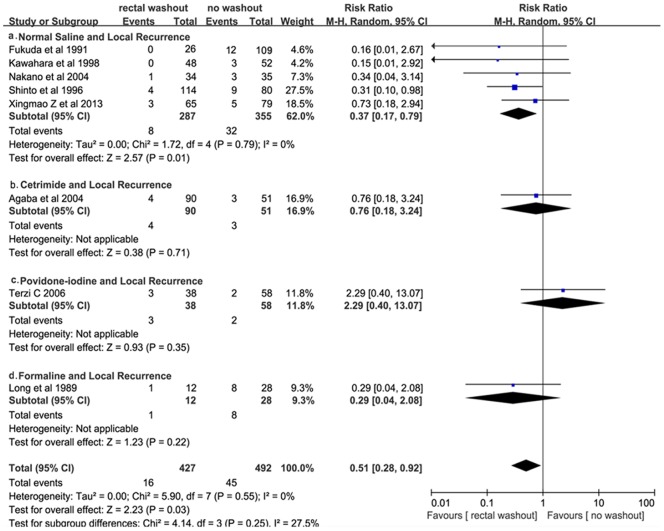
Effect of Rectal Washout on Local Recurrence.

The analysis on the effects of intra-operative rectal washout solutions on local recurrence (LR) status was based on 8 trials or 919 participants. A significant effect of WO in LR status *(16/427, 3.75% vs. 45/492, 9.15%; RR = 0.51, 95%CI = 0.28–0.92, P = 0.03* was showed in *figure 2*, with no statistical heterogeneity *(I^2^ = 0.00, Tau = 0.00, χ2 = 5.90, P = 0.55)*.

*5* studies or *642* participants investigated the effect of normal saline washout on local recurrence (LR), with an overall LR rate of *6.23% (40/642)*. A difference in normal saline washout was showed to have a relevance to the reduction in risk of LR(*8/287, 2.79% vs. 9.01%,32/355; RR = 0.37, 95% CI = 0.17–0.79, P = 0.01),* with no statistical heterogeneity *(I^2^ = 0.00, Tau = 0.00, χ^2^ = 1.72, P = 0.79)*.

The remaining three studies reported data on local recurrence in patients having rectal washout with *1%* cetrimide, *5%* povidone-iodine and *1%* formaline, respectively. Local recurrence occurred in *4.44% (4/90)* or *5.88% (3/51)* of patients having cetrimide washout or not, in *7.89% (3/38)* or *3.45% (2/58)* of patients having povidone-iodine washout or not, in *8.33% (1/12)* or *28.57% (8/28)* of patients having formaline washout or not. No statistically significant differences were detected between the two groups in each pair *(P>0.05)*.

### Association of Washout Volume with Risk of Local Recurrence

**Figure 3:**
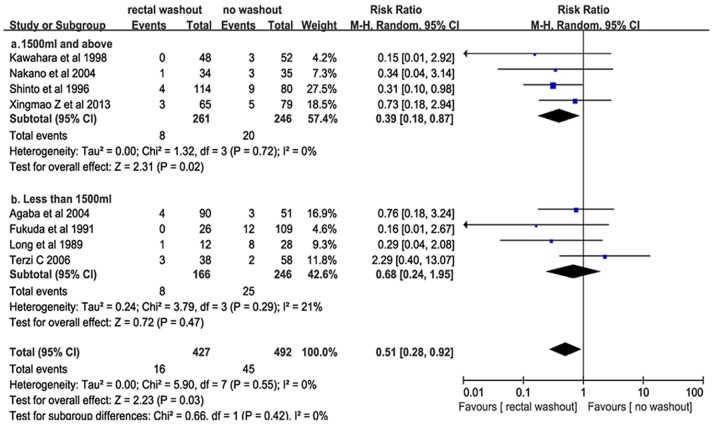
Washout solution volumes and local recurrence.

To study the effect on readmission, data were available for 8 trials or 919 participants, of which 261 participants received 1500 ml and above rectal washout in volume for rectal washout during rectal cancer resection, 246 not, 166 received less than 1500 ml in volume and 246 not, as shown in *figure 3*. A difference in LR in 1500 ml and above subgroup was showed to have a relevance to the reduction in risk of AR (*RR = 0.39, 95% CI = 0.18–0.87, P = 0.02),* with no statistical heterogeneity *(I^2^ = 0.00, Tau = 0.00, χ2 = 1.32, P = 0.72).* But, less than 1500 ml in volume was not associated with a difference in LR (*8/166, 4.82% vs. 16.12%,25/246; RR = 0.68, 95%CI = 0.24–1.95, P = 0.47).* Statistical heterogeneity was low *(I^2^ = 0.21, Tau = 0.24, χ^2^ = 5.79, P = 0.29)*.

### Sensitivity Analysis

Sensitivity analyses *(figure S1)* indicated that three independent studies by Agaba *et al*, Terzi *et al* and Xingmao Z *et al* were the main origin of heterogeneity. The heterogeneity was decreased or vanished after deletion of study by Terzi *et al*, while the association still kept significant except for washout solutions subgroup (*Table 3*). In addition, no other single study influenced the pooled RR qualitatively, as indicated by sensitivity analyses, suggesting that the results of this meta-analysis are stable.

**Table 3:** Results of Meta-analysis after Excluding the Heterogeneous Study.

Subgroup	NO	Group	N	Rate (%)	Heterogeneity			Excluding corresponding study or not					
					*χ2*	*I^2^ (%)*	*P*	no			yes		
								*RR*	*95% CI*	*P*	*OR*	*95% CI*	*P*
***WO and LR***	8	*WO*	427	6.64	6.13	0	0.53	0.44	0.24–0.80	0.007	NA	NA	NA
		*NOW*	492										
*Saline*	5	*WO*	287	6.23	1.74	0	0.78	0.32	0.15–0.70	0.004	NA	NA	NA
		*NOW*	355										
*Cetrimide*	1	*WO*	90	4.96	NA	NA	NA	0.74	0.16–3.46	0.71	NA	NA	NA
		*NOW*	51										
*Povidone-iodine*	1	*WO*	38	5.21	NA	NA	NA	2.4	0.38–15.09	0.35	NA	NA	NA
		*NOW*	58										
*Formaline*	1	*WO*	325	22.5	NA	NA	NA	0.23	0.03–2.06	0.19	NA	NA	NA
		*NOW*	413										
***WV and LR***	7	*WO*	389	6.41	2.61	0	0.86	0.51	0.28–0.92	0.03	0.35	0.22–0.78	0.007
		*NOW*	434										
*1500* *ml and above*	4	*WO*	261	5.52	1.32	0	0.72	0.39	0.18–0.87	0.02	NA	NA	NA
		*NOW*	246										
*less than 1500* *ml*	3	*WO*	166	8.01	1.29	0	0.52	0.68	0.24–1.95	0.47	0.45	0.15–1.33	0.15
		*NOW*	246										

WO = washout group; NWO = no washout group; LR: local recurrence; AR: anastomotic recurrence; WV: washout volume; NO: serial number of a study N: the number of patients; rate: rate of events; NS = not stated; Rate: LR rate or AR rate; RR :risk ratio; OR: odds ratio.

### Publication Bias Analysis

Begg’s funnel plot *(figure S2)* and Egger’s test *(* *Table 4* *)* were performed to assess publication of included studies. The shapes of funnel plot did not reveal any evidence of obvious asymmetry in all genetic models. Egger’s test, which was applied to provide statistical evidence of funnel plot symmetry, did not indicated asymmetry of the funnel plot *(P = 0.886)*, suggesting that no significant publication bias was found although significant heterogeneity between these studies was observed.

**Table 4:** Egger’s test of 8 Included Studies.

Std_Eff	Coef.	Std. Err.	t	P>|t|	[95% Conf.	Interval]
slope	0.808026	0.811462	1	0.358	−1.17755	2.793601
bias	−0.14036	0.937052	−0.15	0.886	−2.43325	2.15252

## Discussion

Theoretically, post-operative local recurrence may occur for several reasons. In a majority of cases a prerequisite is that viable tumor cells remain in the pelvis after surgery [12], [13], [43]. This can occur in the following two principal ways: i)solid tumor tissue is left behind (for example in remaining bowel wall, mesorectum, pelvic side walls or lateral lymph nodes), ii)free viable tumor cells are left in the surgical field and implant (for example spillage from peritumoral perforations [44] or from the transected rectal lumen, or incorporation into the anastomosis [41]. We need to acknowledge that remaining solid tumor tissue has shrunk or disappear due to current advances in rectal cancer, such as stapling technique [45], [46], and neoadjuvant chemoradiotherapy [47]. Therefore, the remaining way was assumed to have relevance to the risk or local recurrence for rectal cancer owing to the viability of shed intraluminal cells was previously established and malignant cells were retrieved on circular staplers in unwashed rectal stumps. Furthermore, the speculation about had been confirmed by a series of in vitro and animal experiments. For instance, viable exfoliated tumor cells were demonstrated in 70%(52/74) specimens by Umpleby and colleagues [13], proven to be viable and capable of growth in vitro by Skipper and colleagues [43], and could result in mucosal implantation and intraluminal tumor growth after damaged the colon mucosa of the rats [48]. Then, intraoperative spillage of tumor cells were confirmed to influence the incidence of local recurrence, even after having total mesorectum excision [49],[50]. Consequently, rectal washout has the potential to eradicate free malignant cells shed into the rectal stump during anterior resection because of the mechanical washing or tumoricidal agents contained in the washout fluids [5], [14]–[16].

In our meta-analysis, the pooled RR *0.51(95% CI = 0.28–0.92, P = 0.03)* indicated that application of rectal washout resulted in a statistically significant reduction of LR. The outcome was similar to those by Rondelli and colleagues [26] as well as Matsuda and colleagues [34]. But the RR value was lower than 0.57∼0.64 in the latter two studies. The differences may own to the fact that none of these previous meta-analyses included the studies published in 2013. In addition, the meta-analyses by Rondelli and colleagues [26] and Matsuda and colleagues [34] included the Kodada’s study [41] while the Kodada’s study was not included in our meta-analysis.

In clinical work, we know that rectal washout can be fall into two categories according to the containing agents in the irrigation fluids: non-tumoricidal agents (such as normal saline[18], [22]–[25]) and tumoricidal agents (such as cetrimide [17], povidone-iodine [21], and formalin [19]). Normal saline solution (0.9% w/v of NaCl), also known as 0.9% NaCl or physiological saline, had the same osmotic pressure as human plasma and has no damaging effect on normal cells or tumor cells, or no tumoricidal effect, and became one of the most common solutions used in cytological study. The subgroup analysis in our study, the pooled RR *0.37 (95% CI = 0.17–0.79, P = 0.01 )* in the normal saline group, reveals that rectal washout with normal saline alone can significantly reduce the risk of local recurrence of rectal cancer by 63%. The reason may be that exfoliated malignant cells can be mechanically removed from the distal rectum by rectal washout, primarily through mechanical cleansing, rather than any cytocidal effect of the irrigation fluid as was previously thought [14], [15]. However, there was a lack of power to show statistical significance in results when it comes to cetrimide, povidone-iodine, or formalin solution in our meta-analysis, with the reasons that the included studies had small or few events (local recurrences) or blood made povidone-iodine and cetrimide less efficient at killing colorectal cells [41]. It is impossible therefore to quantify contribution made by the lavage cetrimide, povidone-iodine or formaline. Moreover, we could not distinguish which washout solution was most effective at lowering local recurrence rates, irrespective of the serious complications induced by cytocidal solutions, such as cetrimide and chlorhexidine [51].

As before-mentioned, clinical evidence has demonstrated the completeness of cleansing with irrigation fluid is volume-related in eradication of intraluminal malignant cells during anterior resection [14], [28]. Sayfan and colleagues demonstrated that the effectiveness of washout by physiological solution depends on irrigation volumes, such that *1.5 L* was required for tumors below the peritoneal reflection and *2 L* for those above the peritoneal reflection [28]. Therefore, *1.5 L* interested us and was used as volume boundary. In our analysis, the RRs *0.39 (95% CI = 0.18–0.87, P = 0.02)* in the 1500 ml and above subgroup and *0.68 (95% CI = 0.24–1.95, P = 0.47)* reveal that the application of *1.5 L* irrigation fluid can reduce the risk of local recurrence for rectal cancer. The underlying mechanism from mechanical cleaning follows as: exfoliated malignant cells i) can be mechanically removed from the distal rectum and then resorpted by vacuum extractor, ii) flushed with irrigation fluid from damaged colon mucosa and serosa to intact areas, which results in mucosal implantation and intraluminal tumor growth due to the resistance to implantation through intact mucosa and serosa. But no matter the reason, the research shows that a thorough rectal irrigation will probably eliminate exfoliated malignant cells, and that a minimum of 1.5 liters of normal saline is recommend for rectal washout during rectal resection.

Nevertheless, the following limitations should be taken into consideration when the results of this study are interpreted. First of all, the relation may not necessarily be causal [52], because of possible confounding factors such as the treatment, or the characteristics of the tumor (such as pathologic type, differentiation degree, vascular invasion, lymphatic vessel invasion status, lymph node status, margin and operation type, or TNM-stage) [53]–[57]. For this reason, there still exists different opinions concerning whether rectal washout using a minimum of1.5 liters of normal saline during anterior resections for cancers is an independent prognostic factor for LR.

Secondly, the eight included studies are not randomized controlled trials (RCTs) but non-randomized case-control studies. RCTs provide better evidence for potential treatment effects/harms than non-randomized case-control studies. In the meta-analysis of the eight studies, the individual and pooled estimates comparing the preventive effects of different irrigation fluid types and volumes were not neutral [58]. But, we are convinced that surgeons will continue to perform this technique until strong evidence suggests otherwise [59], due to the low cost and ease involved.

Thirdly, it is important to note that the populations of the included studies were heterogeneous, most probably because of the diversity of the study designs, ethnic diversity, and lack of standardized protocol, which may result in an overestimation of the effect of rectal washout.

For all these limitations, our analysis supports reduction of LR or AR by rectal washout using 1.5 liters or more than 1.5 liters of normal saline during anterior resection of rectal cancer. However, clinicians should be provided with an additional incentive to pay integrated clinical attention and elucidate the complex interactions between washout types or irrigation volumes and local recurrence of rectal cancer.

## Conclusions

Our meta-analysis favors reduction of LR by rectal washout using a minimum of 1.5 liters of normal saline during anterior resection of rectal cancer. However, since no RCTs included, our findings underscored the need for perspective multicenter randomized studies to confirm this potential benefit.

## Supporting Information

Figure S1 **Sensitivity Analysis Plot of 8 Included Studies.**
(TIF)

Figure S2 **Begger’ Funnel Plot of 8 Included Studies.**
(TIF)

Checklist S1 **PRISMA Checklist.**
(DOC)
