# Supplementary figures and images for: Association between Irrigation Fluids, Washout Volumes and Risk of Local Recurrence of Anterior Resection for Rectal Cancer: A Meta-Analysis of 427 Cases and 492 Controls
Source: PLoS One. 2014 May 13;9(5):e95699. doi: 10.1371/journal.pone.0095699 (PMC4019500; doi:10.1371/journal.pone.0095699)

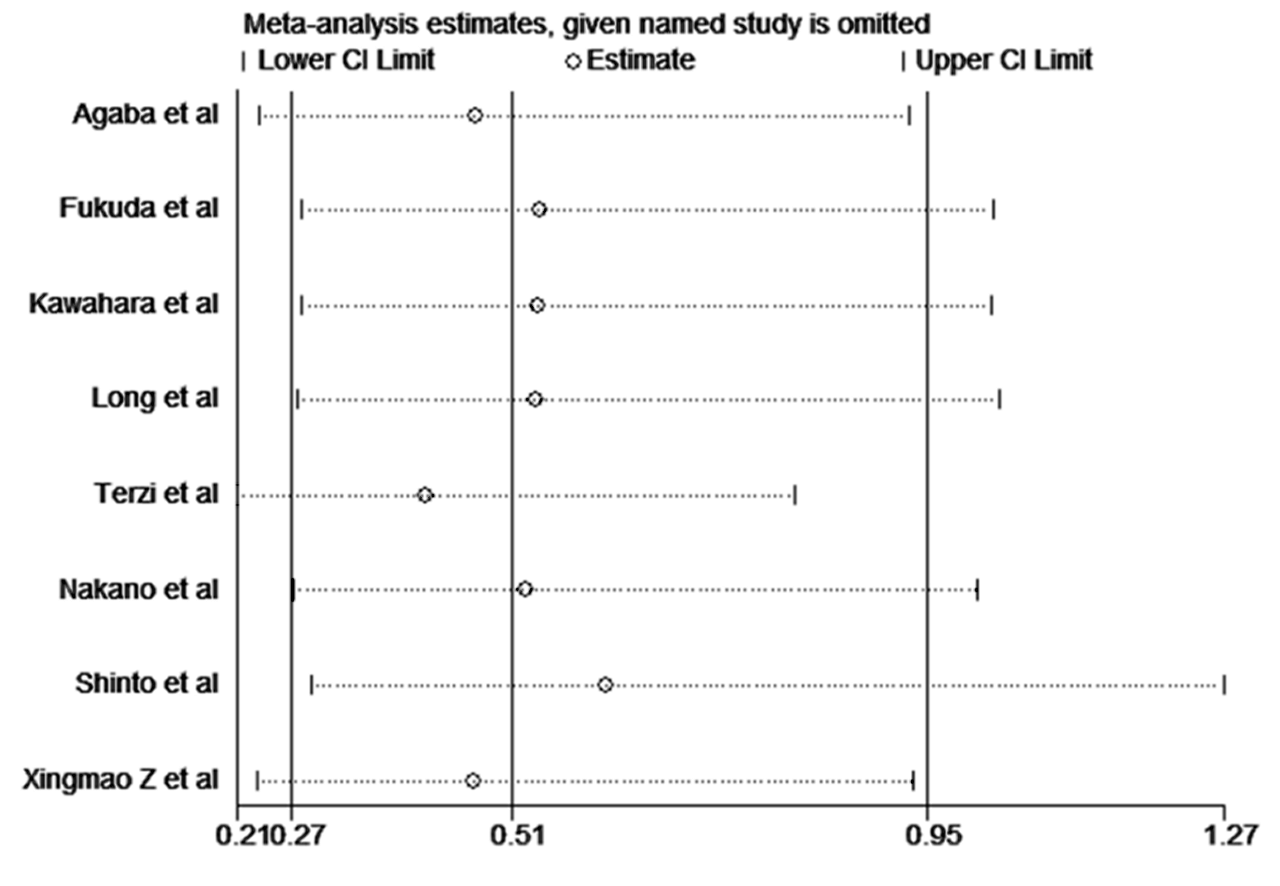

Supplement: Figure S1 — Sensitivity Analysis Plot of 8 Included Studies. (TIF) [file pone.0095699.s001.tif]

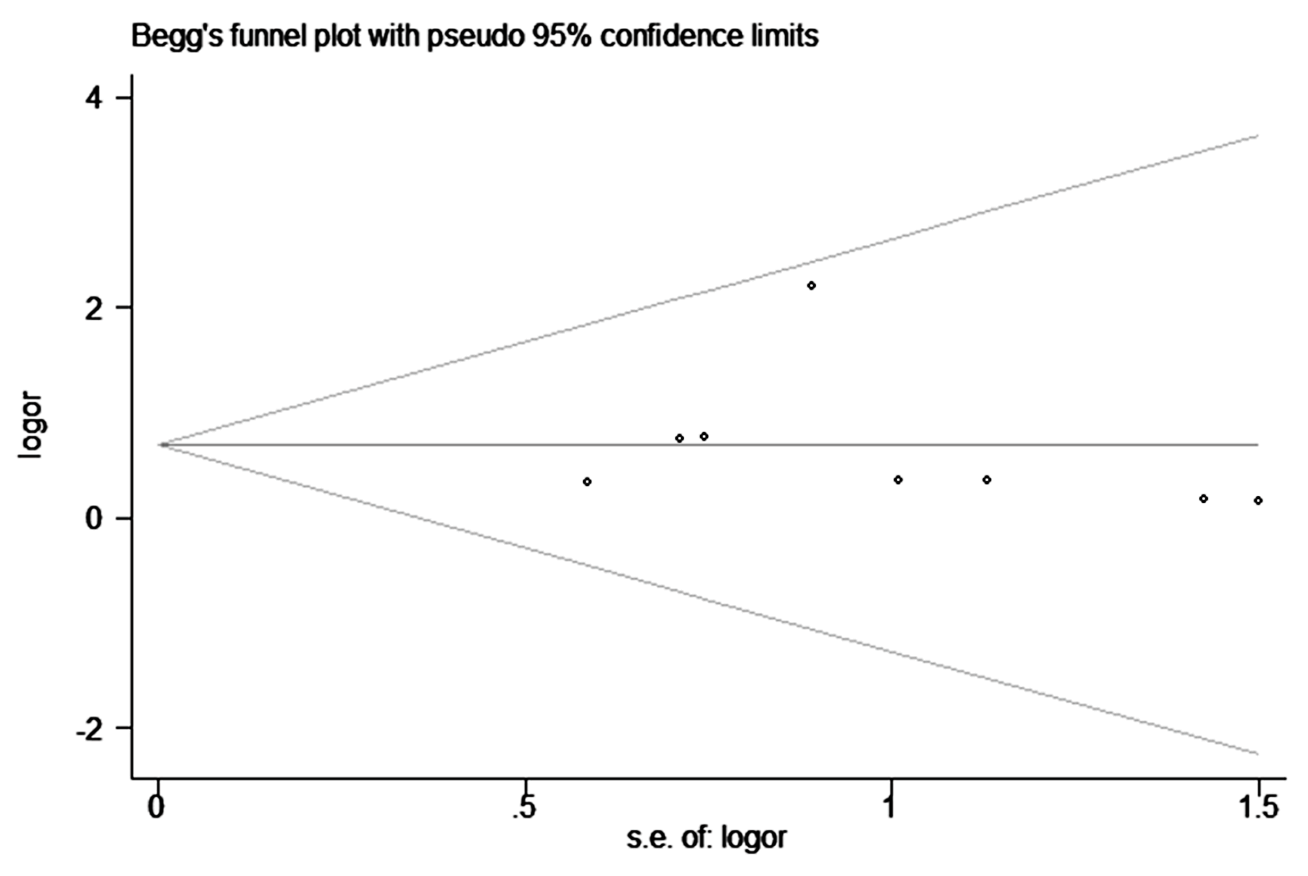

Supplement: Figure S2 — Begger’ Funnel Plot of 8 Included Studies. (TIF) [file pone.0095699.s002.tif]
